# Reproducible Microstructural Changes in the Brain Associated With the Presence and Severity of Urologic Chronic Pelvic Pain Syndrome (UCPPS): A 3-Year Longitudinal Diffusion Tensor Imaging Study From the MAPP Network

**DOI:** 10.1016/j.jpain.2022.11.008

**Published:** 2022-11-23

**Authors:** Chencai Wang, Jason J. Kutch, Jennifer S. Labus, Claire C. Yang, Richard E Harris, Emeran A. Mayer, Benjamin M. Ellingson

**Affiliations:** * Department of Radiological Science, David Geffen School of Medicine, University of California Los Angeles, Los Angeles, California; † Division of Biokinesiology and Physical Therapy, University of Southern California, Los Angeles, California; ‡ Oppenheimer Center for the Neurobiology of Stress and Resilience, David Geffen School of Medicine, University of California Los Angeles, Los Angeles, California; § Vatche and Tamar Manoukian Division of Digestive Diseases, Department of Medicine, David Geffen School of Medicine, University of California Los Angeles, Los Angeles, California; ¶ Department of Psychiatry and Biobehavioral Sciences, David Geffen School of Medicine, University of California Los Angeles, Los Angeles, California; ‖ Department of Urology, University of Washington, Seattle, Washington; ** Chronic Pain and Fatigue Research Center, Department of Anesthesiology, University of Michigan, Ann Arbor, Michigan

**Keywords:** Urological chronic pelvic pain, UCPPS, Longitudinal observation, Diffusion tensor imaging, Probabilistic tractography

## Abstract

Microstructural alterations have been reported in patients with urologic chronic pelvic pain syndrome (UCPPS). However, it isn’t clear whether these alterations are reproducible within 6 months or whether long-term symptom improvement is associated with specific microstructural changes. Using data from the MAPP-II Research Network, the current study performed population-based voxel-wise DTI and probabilistic tractography in a large sample of participants from the multicenter cohort with UCPPS (N = 364) and healthy controls (HCs, N = 61) over 36 months. While fractional anisotropy (FA) differences between UCPPS patients and HCs were observed to be unique at baseline and 6-month follow-up visits, consistent aberrations in mean diffusivity (MD) were observed between UCPPS and HCs at baseline and repeated at 6 months. Additionally, compared to HCs, UCPPS patients showed stronger structural connectivity (SC) between the left postcentral gyrus and the left precuneus, and weaker SC from the left cuneus to the left lateral occipital cortex and the isthmus of the left cingulate cortex at baseline and 6-month. By 36 months, reduced FA and MD aberrations in these same regions were associated with symptom improvement in UCPPS. Together, results suggest changes in white matter microstructure may play a role in the persistent pain symptoms in UCPPS.

Urological chronic pelvic pain syndrome (UCPPS), an umbrella term for the diagnoses of chronic prostatitis/chronic pelvic pain syndrome (CP/CPPS) and interstitial cystitis/bladder pain syndrome (IC/BPS), is characterized by persistent pain and discomfort perceived to be related to the bladder or pelvic region.^2,20^ Clinical and experimental efforts have been made to better understand the pathophysiology of UCPPS, and recent studies have suggested that potential combination of both peripheral and central nervous system biomarkers may be the best approach to phenotyping, diagnosis, and treatment.^3,8,22,26,36^

The Multidisciplinary Approach to the Study of Chronic Pelvic Pain (MAPP) Research Network was created in part to identify brain imaging biomarkers that are sensitive to diagnosis or early progression of UCPPS and to evaluate treatment effectiveness.^1,8,9^ Specifically, using diffusion tensor imaging (DTI) indices such as the fractional anisotropy (FA), which reflects the directionality of diffusion, the mean diffusivity (MD), which reflects the tissue microstructure, and fiber track density (TD), previous studies have reported extensive microstructural differences within the brain of UCPPS patients.^14,15,22,26–28,36^ For example, compared to HCs, UCPPS patients showed lower FA, higher MD and lower TD along corpus callosum, superior and inferior longitudinal fasciculi, frontal/prefrontal projects, and white matter tracts connecting the cingulate, parietal and temporal lobes. Additionally, group differences in cerebral DTI measures were positively correlated with pain and symptom severity scores, including segments of the right corticospinal tract, the right anterior thalamic radiation, as well as within regions of the sensorimotor network and cingulate cortical areas. However, the extent to which these differences are reproducible in a separate, independent cohort of patients using multiple measurements, as well as how any fluctuations in imaging measurements might reflect UCPPS symptom progression, remains largely unknown.

Since UCPPS has been characterized as a chronic disease with no significant changes in overall disease severity,^31,32^ we hypothesize DTI differences between UCPPS and HCs inherent to the disease could be identified and reproduced when looking at measurements over a short period of (ie, 6 months). Additionally, we studied DTI changes in UCPPS occurring within these distinct areas shown to be uniquely altered in UCPPS patients during long-term (i*e,* 3 year) changes in UCPPS clinical symptoms. To test these hypotheses, differences in white matter integrity between UCPPS patients and HCs were evaluated twice, 6 months apart, to determine “disease-associated” fractional anisotropy (FA), mean diffusivity (MD), and microstructural connectivity (SC) measured using probabilistic tractography. We theorize that, despite any transient fluctuations in symptoms, white matter microstructural reorganization is not likely to occur within a short 6-month interval. Then, we examined long-term, 36-month fluctuations in symptom severity and quantified FA, MD, and microstructural connectivity changes that correlate with these fluctuations, then determined whether these areas overlap spatially in the brain with “disease-associated” patterns observed when comparing UCPPS and HCs. In this case, we theorize white matter reorganization, if it does occur, would manifest over a longer period of time (~3 years).

## Methods

### Patients

A total of 364 UCPPS participants (132 men, 232 women, average age = 44.1 ± 15.5 years) and 61 heathy controls (HCs, 28 men, 33 women, average age = 41.8 ± 14.7 years) were prospectively enrolled in the current study (MAPP-II) that included observational multimodal MRI and evaluation of UCPPS symptoms. Inclusion criteria for UCPPS participants consisted of^9,29^: 1) UCPPS symptoms present for a majority of the time during most recent 3 months, 2) age 18 years and above, and 3) response 1 or greater on the bladder/prostate or pelvic pain/pressure/discomfort scale (0–10 scale)^12,17,35^ during past 2 weeks. Exclusion criteria include symptomatic urethral stricture, neurological disease or disorder affecting the bladder, bladder fistula, a history of cystitis caused by tuberculosis, radiation therapy or chemotherapy, prior augmentation cystoplasty or cystectomy, active autoimmune or infectious disorder, history of pelvic cancer, current major psychiatric disorder, severe cardiac, pulmonary, renal, or hepatic disease, unilateral orchalgia (without pelvic symptoms), and prior prostate procedures (transurethral microwave thermo-therapy, transurethral needle ablation, balloon dilation, prostate cryosurgery, or laser procedure). Participants from the HC cohort were excluded if they reported any pain in the pelvic or bladder region. For more specific inclusion and exclusion criteria, please see Clemens et al.^7^ All subjects provided informed written consent to participate in the current study. All consenting procedures and protocols were approved by the institutional review board at each of the participating sites, which included the University of California, Los Angeles (UCLA), Northwestern University (NWU), University of Michigan (UM), University of Iowa (UI), University of Washington (UW), and Washington University in St. Louis (WUSTL). Detailed demographic for all participants and for participants of each site were summarized in Table 1, and Table S1 (Supplementary Table), respectively.

### Experimental Design

**Figure 1.**
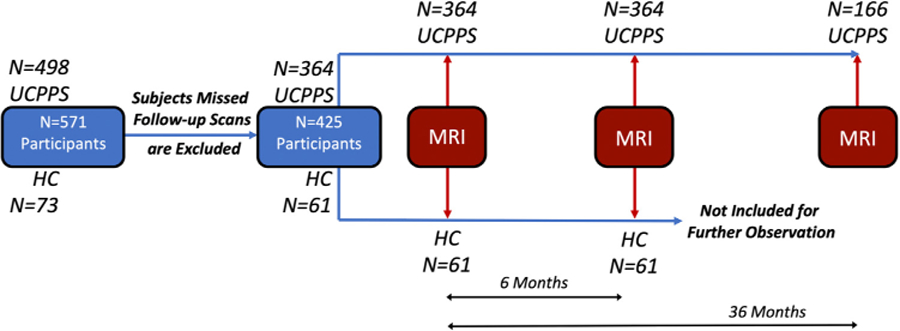
Schematic illustrating the MAPP2 DTI trial design.

Neuroimaging was conducted during multiple visits that took place within 4 weeks of the baseline visit, 6-month follow-up, and 36-month follow-up (Fig 1).^29^ Multiple urologic symptom measures previously included in the MAPP I symptom patterns study protocol^29^ were retained in the current longitudinal observation. After organizing all UCPPS outcomes based on pain and urinary symptoms, an exploratory factor analyses within the MAPP research network^19^ identified 2 unique dimensions to UCPPS based on pain (0–28 scale) and urinary (0–25 scale) severity. This group then assigned composite pain and urinary symptom scores based on combinations of these symptom measures (eg, Genitourinary Pain Index [GUPI], Interstitial Cystitis Symptom Index [ICSI], the Questionnaire for Bladder Pain, and Symptom and Health Care Utilization Questionnaire [SYMQ] item 4) as outlined in Griffith et al.^19^ Note the focus of the current study was to explore the impact of composite measures of pain severity on changes in white matter integrity only, so urinary symptom severity was not explicitly examined. Detailed clinical data for all participants and for participants of each site were summarized in Table 1, and Table S1 (Supplementary Table), respectively.

### DTI Data Acquisition

MRI scanning was performed at multiple sites using different scanner technology (3T Siemens Prisma (UCLA and UI), 3T GE Discovery (UM), 3T Philips Achieva (UW), and 3T Siemens Trio (NWU and WUSTL)). Neuroimaging data were collected, quality controlled, and archived according to multi-site imaging procedures developed collaboratively between the MAPP Research Network, the UCLA PAIN repository, and the UCLA Laboratory of Neuroimaging. Detailed procedures and descriptions of the repository are available at PAINrepository.org.

The acquisition parameters for DTI at different scanning sites were summarized in Table 2. Diffusion weighting was distributed along 64 equidistant directions using a *b*-value of 1000 s/mm^2^. Additionally, a single reference *b=0* s/mm^2^ image was used as a reference and a 1 mm 3D isotropic MPRAGE T1-weighted image was acquired for alignment. The acquisition parameters for T1-weighted images at different scanning sites are also summarized in Table 2.

### Image Processing

All DTI data were quality controlled according to multi-site imaging procedures developed within the MAPP Research Network. Image processing and analyses of the DTI data were conducted at the UCLA Center for Computer Vision and Imaging Biomarkers. All diffusion-weighted images were first denoised using the *MRtrix3* software package (Brain Research Institute, Melbourne, Australia, http://www.brain.org.au/software/mrtrix).^6^ Then FMRIB’s Diffusion Toolbox (FDT) (https://fsl.fmrib.ox.ac.uk/fsl/fslwiki/FDT) was used to correct for eddy currents (which cause stretches and shears in images) and head motion, and images were affinely registered to reference *b* = 0 s/mm^2^ images for each participant. Following skull extraction using the Brain Extraction Tool (BET), fractional anisotropy (FA) and mean diffusivity (MD) values at the voxel level were calculated for DTI, using mask consisting of the disjunction between the white matter and deep gray matter masks of interest (namely the basal ganglia and thalamus, defined by their respective masks from the Harvard-Oxford subcortical atlas) as defined previously.^13^ To identify and anatomically localize group differences between UCPPS and HCs in FA and MD, all FA and MD images were linear and nonlinearly registered to the Johns Hopkins University DTI atlas (ICBM-DTI-81 1mm FA atlas) using the FLIRT and FNIRT commands, respectively, in FSL (https://fsl.fmrib.ox.ac.uk/fsl).

Probabilistic DTI tractography was performed using the FMRIB Diffusion Toolbox (FDT & Probtrackx; FMRIB; Oxford, UK; http://www.fmrib.ox.ac.uk/fsl/fdt/). After DTI data for each patient was eddy current corrected, Markov Chain Monte Carlo sampling was used to build the diffusion orientation distribution functions (ODFs) for each voxel using Bedpostx (BEDPOSTX; FMRIB; Oxford, UK; http://www.fmrib.ox.ac.uk/fsl/fdt/) to model a total of 2 fibers per voxel and a total of 1,000 iterations. After the distributions for each diffusion parameter were generated for each patient, seed and target ROIs were placed on anatomical landmarks defined in the Desikan-Killiany cortical atlas to quantify the connectivity between these structures.^10^ A total of 5,000 individual pathways were generated from the seed points using a step length of 0.5mm and a maximum of 2,000 steps. A cosine curvature threshold of 0.2 (approximately 80 degrees) was used to limit how sharply pathways can deflect during tract generation. Additionally, a waypoint mask using common clusters showing differences in either FA or MD at both baseline and 6-month follow-up was used to prune and isolate results to streamlines that pass through the areas in the brain commonly altered in UCPPS relative to HCs. Lastly, ROI-to-ROI SC networks were computed between all brain regions defined in MNI, where the number of streamlines that pass through the waypoint mask represent the weight of the specific regions in the network. Abbreviations for ROIs can be referred to Table S2 (Supplementary Table).

### Image Statistical Analysis

Using preprocessed DTI data from HC cohort, we performed analysis of variance (ANOVA) to decide if statistical correction for site effect will be included when investigating reproducible microstructural changes associated with UCPPS. It is also important to note that because 1) no significant site differences were observed within HC cohorts, and 2) unlike MAPP-I, the DTI acquisition parameters and sampling scheme were standardized for MAPP-II, we decided to not include the site effect as a covariate in all statistical analyses.

To test whether significant differences occurred between baseline and 6 month follow up, repeated measures two-way ANOVAs were performed. A general linear model (GLM), with age and sex included as covariates, was implemented through AFNI (Analysis of Functional NeuroImages, https://afni.nimh.nih.gov/) to 1) evaluate common inter-group differences between UCPPS patients and HCs, at both baseline and 6-month follow-up, and 2) explore the correlation (by Pearson’s correlation coefficient) between changes in DTI measurements and reducing pain symptom severity over longitudinal observation.

To identify inter-group differences in FA and MD between UCPPS patients and HCs, a voxel-wise GLM was utilized through AFNI *3dttest++* command, with age and sex included as covariates. For permutation testing used to estimate the proper cluster threshold, we implemented a family-wise error (FWE) correction approach at the cluster level through command *3dClustSim* in AFNI. The cluster-extent threshold corresponded to the statistical probability (*α* = .05, or 5% chance) of identifying a random noise cluster at a predefined voxel-wise threshold of *P* < .05 (uncorrected). This process was performed individually for both baseline and 6-month follow-up comparisons. After exporting cluster masks at both time points, the masks were superimposed to identify overlapping clusters with common differences in FA and MD. For SC network comparisons, we performed ROI-to-ROI analyses using a GLM with age and sex included as covariates. The level of significance for all statistical analyses was set at *p* < 0.05.

For the correlation analysis using FA and MD measurements, we firstly calculated brain maps representing the changes in FA and MD for each individual participant (through AFNI *3dcalc* command). Then a voxel-wise GLM was utilized through AFNI *3dttest++* command to identify the correlation between changes in FA and MD, and reduce pain severity, with age included as covariates. For permutation testing used to estimate the proper cluster threshold, we implemented a family-wise error (FWE) correction approach at the cluster level through command *3dClustSim* in AFNI. The cluster-extent threshold corresponded to the statistical probability (*α* = 0.5, or 5% chance) of identifying a random noise cluster at a predefined voxel-wise threshold of *P* < .05 (uncorrected). We further superimposed the cluster map on the Johns Hopkins University DTI atlas to identify clusters’ anatomical localization, and the overlapping volume of each cluster with specific pre-defined white matter pathway in the standard space. To explore the correlation between SC network changes and reduced pain severity, we performed ROI-to-ROI analyses with age included as a covariate. The level of significance was set at *P* < .05.

## Results

### Description of Clinical Sample

**Figure 2.**
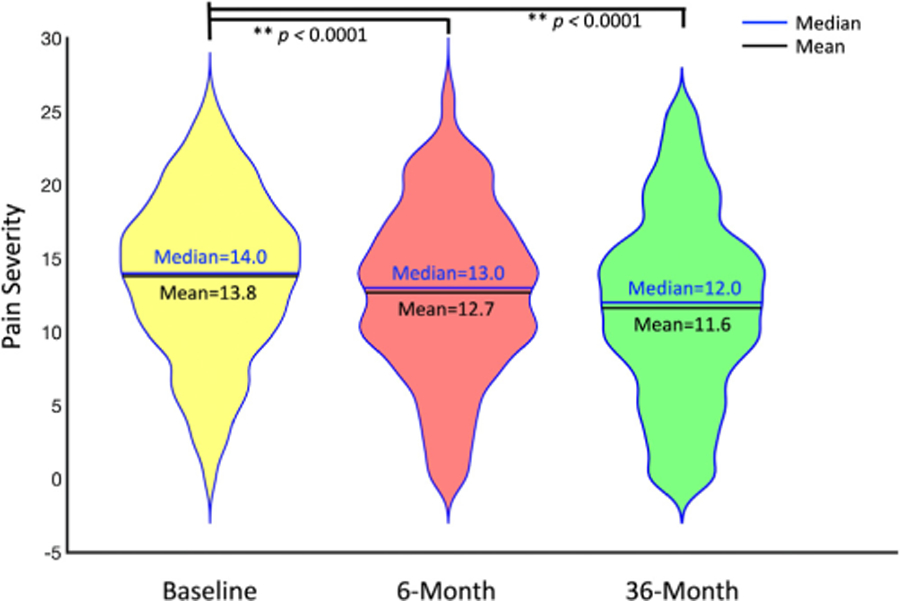
Longitudinal changes of composite pain severity at each time point compared to baseline measurements.

Patient and HC controls cohort did not differ on age (*P* = .2473) or sex (*P* = .1511). When evaluating UCPPS patients pain severity at baseline, 6-month follow-up and 36-month follow-up, respectively, we found a continuous and significant decrease in pain at each clinical visit that was 6 months (mean = 12.7, median = 13.0, *P* < .0001), and 36 months (mean = 11.6, median = 12.0, *P* < .0001) apart from baseline (mean = 13.8, median = 14.0, Fig 2).

### Common Disease-Associated White Matter Alterations in UCPPS

**Figure 3.**
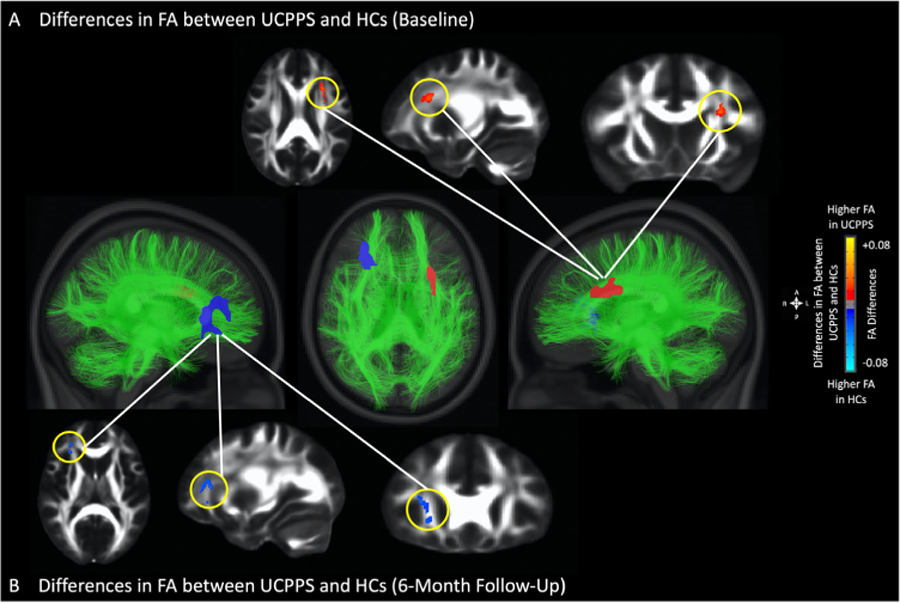
Anatomical localization of significant differences in fractional anisotropy (FA) between UCPPS patients and HCs (A) at baseline (axial view), and (B) at 6-month follow-up (axial view). Significant areas were determined by thresholding based on level of statistical significance (*P* < .05).

**Figure 4.**
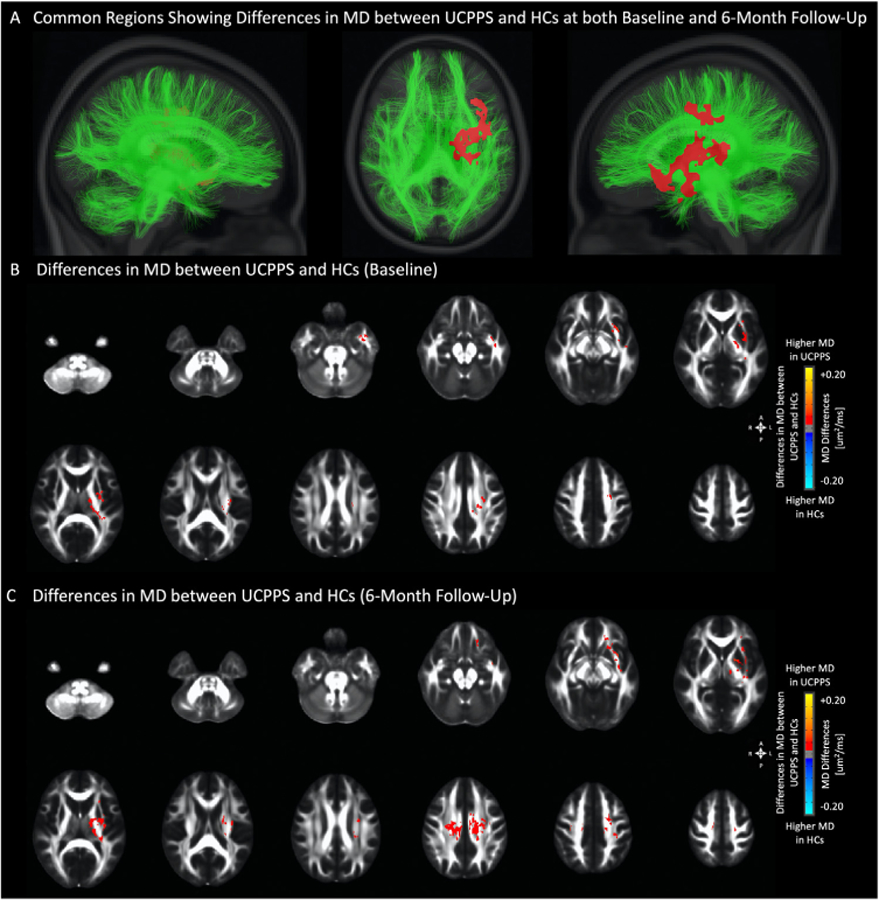
Anatomical localization of significant differences in mean diffusivity (MD) between UCPPS patients and HCs (A) at both baseline and 6-month follow-up (projected onto representative white matter fiber tracts), (B) at baseline (axial view), and (C) at 6-month follow-up (axial view). Significant regions were determined by thresholding based on level of statistical significance (*P* < .05).

To identify *reproducible* microstructural changes associated with UCPPS, or “disease-associated” differences, we compared regional white matter differences characterized by FA and MD at both baseline and a 6-month follow-up. While results from the current study didn’t identify brain regions exhibiting differences in FA at the 2 time points (baseline and 6 months), suggesting no common “disease-associated” FA alterations in UCPPS, several brain regions showed consistently higher MD in UCPPS patients, suggesting these areas might represent reproducible, “disease-associated” brain alterations in UCPPS. Specifically, UCPPS patients exhibited higher FA along the left superior longitudinal fasciculus, the left anterior and superior corona radiata at baseline visit (Fig 3A), but after repeating this comparison 6 months later, results identified lower FA within the right external capsule and along the right corona radiata (Fig 3B and Table 3). MD, on the other hand, was significantly higher in UCPPS patients within the thalamus, the body of the corpus callosum, the left internal (posterior limb and retrolenticular portion) and external capsule, the left corona radiata (superior and posterior regions), as well as the uncinate fasciculus and the superior longitudinal fasciculus in the left hemisphere at both baseline (Fig 4A–B) and 6-month follow-up. (Fig 4A–C and Table 4).

**Figure 5.**
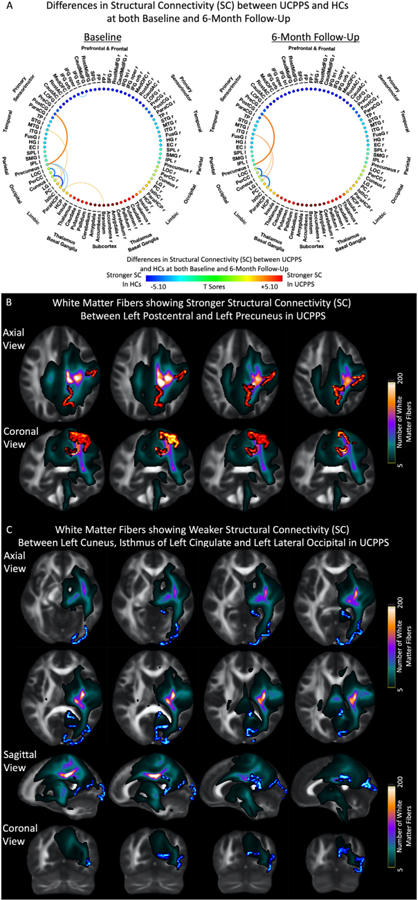
(A) Differences in structural connectivity (SC) between UCPPS patients and HCs at both baseline and 6-month follow-up by constraining streamlines to pass through common regions. Red-Yellow denotes stronger SC in UCPPS patients, while Blue-Light Blue denotes stronger SC in HCs. (B) White matter fibers showing stronger structural connectivity (SC) between the left postcentral gyrus and the left precuneus in UCPPS patients. (C) White matter fibers showing weaker structural connectivity (SC) between the left cuneus, isthmus of the left cingulate and the left lateral occipital cortex in UCPPS patients. Abbreviations: PostCG, Postcentral Gyrus; LOC, Lateral Occipital Cortex; IsthC, Isthmus of Cingulate Gyrus; CaudMidFG, Caudal Middle Frontal Gyrus; IFG oper, Inferior Frontal Gyrus, Pars Opercularis; STG, Superior Temporal Gyrus; MTG, Middle Temporal Gyrus; ITG, Inferior Temporal Gyrus; LG, Lingual Gyrus; PC, Posterior Cingulate Cortex; ParaHCP, Parahippocampus; HCP, Hippocampus; r, Right Hemisphere; l, Left Hemisphere.

By constraining tractography to the thalamic, corpus callosum and other areas exhibiting common elevated MD in UCPPS patients, ROI-to-ROI probabilistic tractography identified repeatable and specific structural connectivity differences between UCPPS patients and HCs (Fig 5A). In particular, UCPPS patients showed stronger connectivity between the left postcentral gyrus and the left precuneus (Fig 5B) at both baseline and 6-month follow-up visits, as well as between the temporal and occipital lobes, but weaker connectivity between the left cuneus to the left lateral occipital cortex and the isthmus of the left cingulate cortex (Fig 5C). Additionally, stronger connectivity was observed between the left para hippocampus and brainstem, while lower connectivity was observed between the left precuneus, the isthmus of the left cingulate cortex and the left para hippocampus in UCPPS patients at the baseline visit, but were not observed at 6-month follow-up.

### Microstructural Changes Associated with Pain Severity Changes at 36 Month Follow-Up

**Figure 6.**
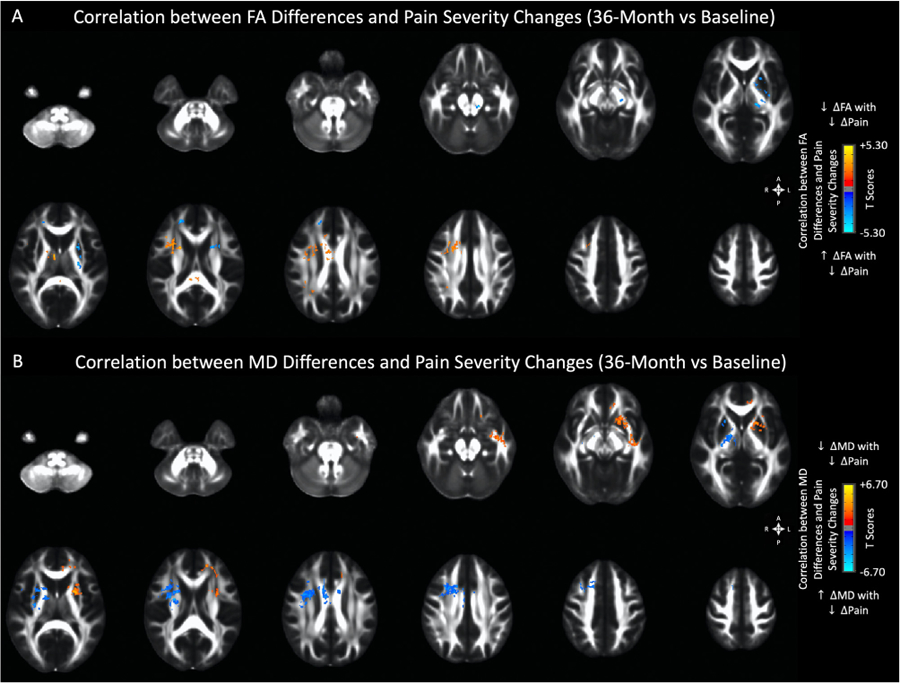
Anatomical localization of clusters showing correlation (A) between fractional anisotropy (FA) differences and pain severity changes at 36-month follow-up, and (B) between mean diffusivity (MD) differences and pain severity changes at 36-month follow-up. Red-Yellow denotes positive correlation (decreased FA or MD with reducing pain) in UCPPS patients, while Blue-Light Blue denotes negative correlation (increased FA or MD with reducing pain) in UCPPS patients. Significant regions were identified by thresholding based on level of statistical significance (*P* < .05).

**Figure 7.**
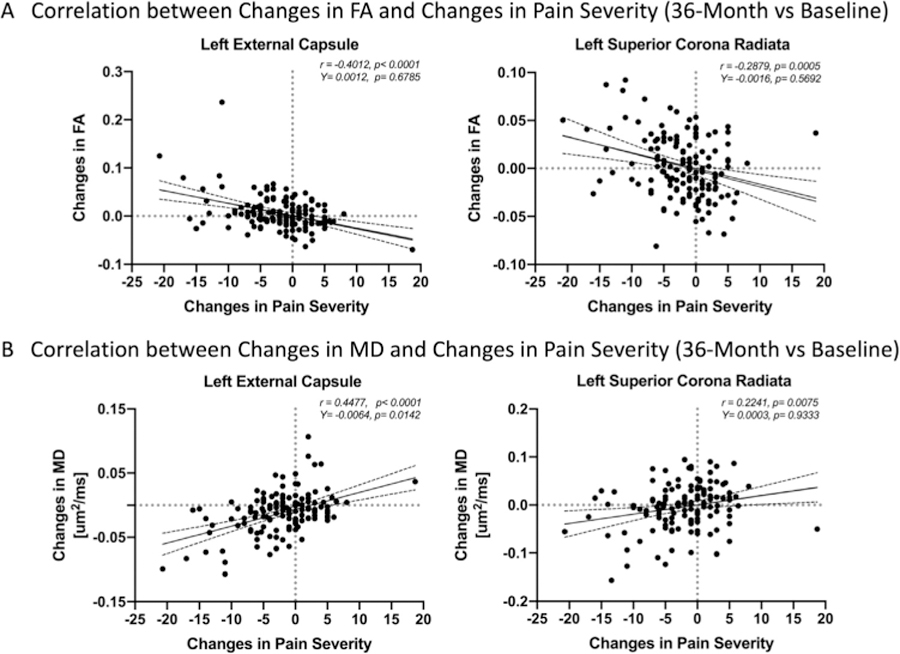
(A) Representative regions, including the left external capsule (r = −.4012, *P* < .0001) and the left superior corona radiata (r = −.2879, *P* = .0005), demonstrating correlation between fractional anisotropy (FA) changes and pain severity changes at 36-month follow-up. (B) Representative regions, including the left external capsule (r = .4477, *P* < .0001) and the left superior corona radiata (r = .2241, *P* = .0075), demonstrating correlation between mean diffusivity (MD) differences and pain severity changes at 36-month follow-up.

**Figure 8.**
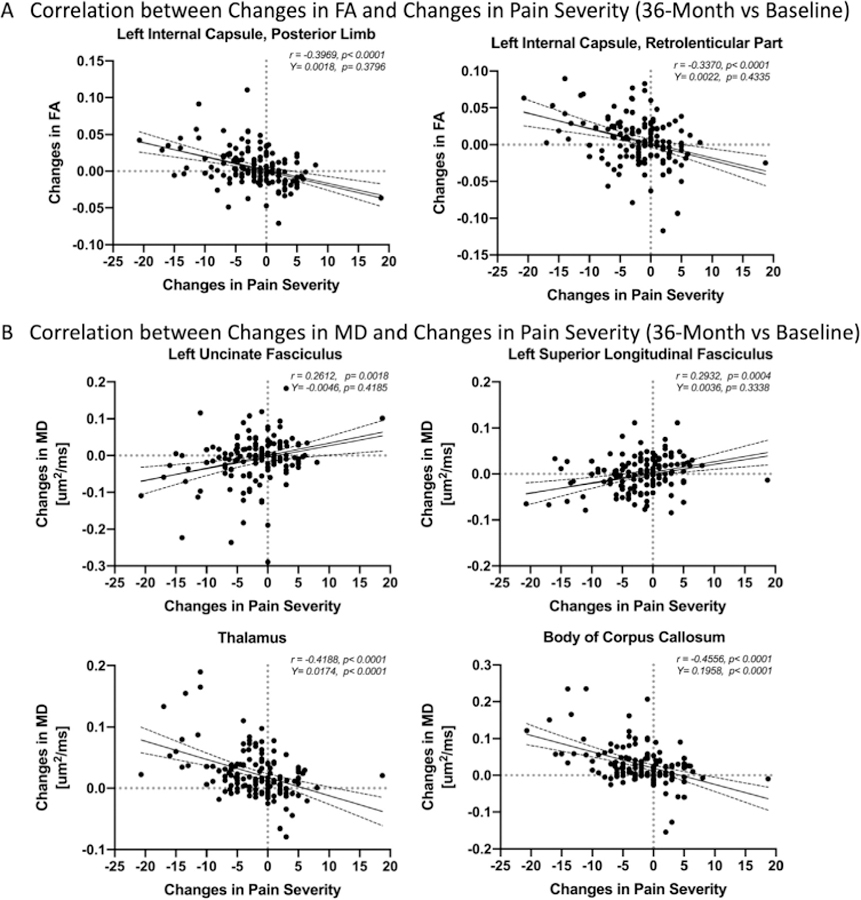
A) Representative clusters, including the posterior limb (r = −.3969, *P* < .0001) and retrolenticular part (r = −.3370, *P* <.0001) of the left internal capsule, demonstrating correlation between fractional anisotropy (FA) changes and pain severity changes at 36-month follow-up. B) Representative regions, including the thalamus (r = −.4188, *P* < .0001) and the body of corpus callosum (r = −.4556, *P* < .0001), demonstrating correlation between mean diffusivity (MD) differences and pain severity changes at 36-month follow-up.

Multiple brain regions, particularly in the areas identified as “disease-associated,” were found to exhibit a significant association between white matter DTI measurements and changes in pain severity when evaluated over 36 months (Fig 6 and Table 5). Among these regions were the left external capsule (Fig 7A; r = −.4012, *p*<0.0001; *Y-intercept* = .0012, *P* = .6,785; Fig 7B; r = .4477, *P* < .0001; *Y-intercept* = −.0016, *P* = .5,692) and the left superior corona radiata (Fig 7C; r = −.2879, *P* = .0005; *Y-intercept*=−.0064, *P* = .0142; Fig 7D; r = .2241, *P* = .0075; *Y-intercept* = .0003, *P* = .9,333), which both demonstrated an increased FA and decreased MD associated with reduced pain symptom severity. Additionally, the posterior limb (r = −0.3969, *P* < .0001; *Y-intercept* = .0018, *P* = .3796, Fig 8A) and retrolenticular portion (r = −.3370, *P* < .0001; *Y-intercept* = .0022, *P* = .4335, Fig 8A) of the left internal capsule demonstrated increased FA associated with reduced pain symptom severity, while the left uncinate fasciculus (r = .2612, *P* = .0018; *Y-intercept*=−.0046, *P* = .4185, Fig 8B) and the left superior longitudinal fasciculus (r = .2932, *P* = .0004; *Y-intercept* = .0036, *P* = .3338, Fig 8B) demonstrated decreased MD associated with reduced pain symptom severity. The thalamus (r = −.4188, *P* < .0001; *Y-intercept* = .0174, *P* < .0001, Fig 8B) and the body of corpus callosum (r = −.4556, *P* < .0001; *Y-intercept* = .1958, *P* < .0001, Fig 8B) demonstrated increased MD associated with reduced pain symptom severity. The bilateral cingulum, and the white matter fiber tracts in the right hemisphere demonstrated increased MD, while the left corona radiata and the anterior limb of the left internal capsule demonstrated decreased MD measurements corresponding with reduced pain symptom severity. Also, several white matter regions, including the right internal and external capsules, the right cingulate, as well as white matter fiber tracts along the body and splenium of corpus callosum showed increased MD associated with reduced pain symptom severit., while association fibers demonstrated a relatively complex pattern of both increased and decreased MD associated with a reduction in pain severity.

### Sex-Dependent Microstructural Changes in UCPPS

**Figure 9.**
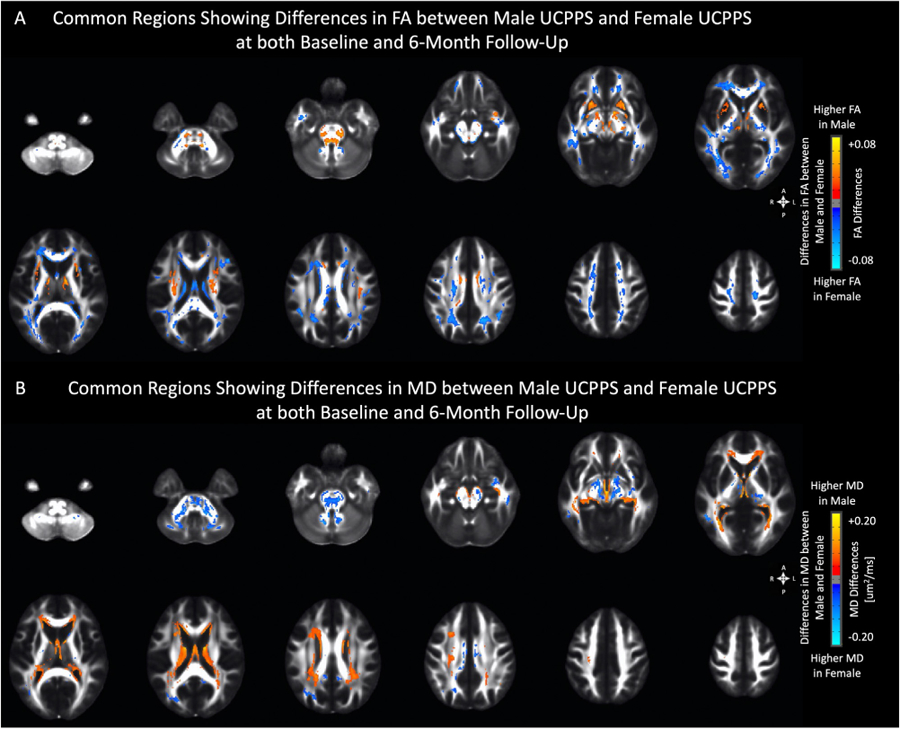
Anatomical localization of clusters showing significant differences in (A) fractional anisotropy (FA) and (B) mean diffusivity (MD) between males and female UCPPS patients at both baseline and 6-month follow-up. Red-Yellow denotes higher FA or MD in male UCPPS patients, while Blue-Light Blue denotes higher FA or MD in female UCPPS patients. Significant regions were identified by thresholding based on level of statistical significance (*P* < .05).

Since male and female patients can differ significantly in terms of their genitourinary symptoms, we next investigated potential sex-dependent microstructural differences between male and female UCPPS patients. While accounting for similar covariates including age, significant sex-dependent differences in DTI measurements were identified between male and female UCPPS patients common to both baseline and 6-month follow up time points (Fig 9). In particular, we observed lower FA within the putamen, thalamus, multiple brainstem regions, and the external capsule, bilaterally, in females with UCPPS, while male patients with UCPPS exhibited significantly lower FA in several large white matter tracts (coronal radiata) projecting to sensorimotor regions as well as tracts projecting to frontal, temporal, and parietal lobe areas both at baseline and 6 months (Fig 9A; Supplemental Fig S1; Supplemental Table S3). Complementary to observations with FA, female UCPPS patients exhibited higher MD within related brainstem and thalamic regions at both baseline and at 6 months (Fig 9B; Supplemental Fig S2; Supplemental Table S4); however, males with UCPPS showed only slightly higher MD along periventricular white matter regions at both baseline and 6-month time points.

## Discussion

### Reproducible MD and SC Alterations Associated with UCPPS

In the current longitudinal study, common “disease-associated” differences in DTI measurements between UCPPS patients and HCs were identified at 2 time points, separated by 6 months. Specifically, results indicated a significantly higher MD in common white matter pathways, including the thalamus, the body of the corpus callosum, the left internal (posterior limb and retrolenticular portions) and external capsule, the left corona radiata (superior and posterior sections), and several association fibers in the left hemisphere known to connect the cingulate, temporal lobes, parietal lobes, sensorimotor areas, and specific frontal/prefrontal regions in UCPPS compared with HCs at both time points. This “disease associated” pattern of altered MD was not only consistently observed in other visceral pain syndrome, eg, irritable bowel syndrome (IBS),^13^ but also reported at a single time point in a separate cohort of UCPPS patients as part of the original MAPP-I research network.^36^ This etiology appears consistent with other studies suggesting structural alteration in the brain may contribute to chronic pain associated with UCPPS^14,15,23,24^ and the notion that UCPPS is a relatively sustained chronic pain condition with only gradual symptom changes over several years and without lasting symptom remission periods.^31,32^

In addition to the aforementioned observations in MD, current study identified reproducible “disease-associated” pattern in SC alterations in UCPPS patients, where stronger SC was observed between the left postcentral gyrus and the left precuneus, and weaker SC from the left cuneus to the left lateral occipital cortex and the isthmus of the left cingulate cortex at both baseline and 6-month follow-up. The precuneus is known to be involved in affective responses to pain,^18^ and the postcentral gyrus, which is the primary sensory region and a main component of the pain matrix. Therefore, a stronger SC between precuneus and the postcentral gyrus might be indicative of alterations in pain perception in chronic stages of disease. Although the lateral occipital cortex was not traditionally associated with brain response to pain, studies have shown that painful stimuli can decrease functional connectivity within the posterior cingulate gyrus (default mode network) and the lateral occipital cortex,^25^ supporting potential hypoconnectivity between the occipital lobe and the isthmus of cingulate cortex in the current study. It should also be noted that altered functional connectivity (FC) among those regions has been reported to be associated with UCPPS patients (MAPP-I research network). Specifically, the posterior medial cortex, including precuneus and the posterior cingulate gyrus, showed decreased FC to default mode network in female UCPPS when comparing to female HCs,^30^ and altered frequency distributions (transformed from BOLD signal) were observed in sensorimotor cortices of UCPPS patients.^24^ The consistency between SC and FC findings suggest that those regions might be potential imaging biomarkers for UCPPS. Together with previous identified “disease-associated” MD differences identified for UCPPS patients with respect to HCs, the current results confirm previous findings of localized alterations in the brain of UCPPS patients,^15,22,36^ and supports the hypothesis that UCPPS is at least partially associated with neuroplastic changes in specific brain areas involved in the processing and integration of sensory inputs and pain modulation, making it potentially amenable for clinical interventions that target synaptic and/or neuronal reorganization (eg, transcranial magnetic stimulation^37^).

### Unique FA Alterations Associated with UCPPS

Differences in FA between UCPPS patients and HCs did not co-localize to the same anatomic regions when comparing scans from baseline and 6 month follow-up. Nevertheless, at both time points, clusters showing significant FA differences were located along similar fiber tracts connecting or projecting to prefrontal/frontal regions and somatosensory processing areas near known pelvic representation. Similar FA alterations have also been reported on the IBS patients,^13^ where lower FA was observed in sensory/motor association/integration regions, but higher FA was observed in frontal lobe regions and the corpus callosum. It is worth noting that since previous study on the separate cohort of UCPPS patients (MAPP-I research network) has suggested that FA changes within these regions might be a consequence of reinforced viscerosensory input to the brain from constant painful stimuli,^36^ implicating an imbalanced altered cortico-basal ganglia-thalamus-cortical loop in pain modulation,^5,11,21^ these alterations did not appear to be “disease-associated,” or commonly altered in all UCPPS patients, as they were not repeatable at the various different time points tested. While these changes do not appear to be commonly altered in *all* UCPPS patients at all time points evaluated, Fig 9 suggest these microstructural alterations in the basal ganglia may be sex dependent. Results from the current study suggest males with UCPPS have significantly higher FA within the basal ganglia when compared with female UCPPS patients, and these changes appear repeatable. Similar sex-dependent alterations in the basal ganglia also appear consistent with a recently published study in a large cohort of IBS patients by Labus et al (Labus, *Pain,* 2022), which demonstrated a significantly higher FA within the basal ganglia in men with IBS, as well as an increase in FA proportional to increased symptom severity. Together, this suggests the potentially altered basal ganglia microstructure observed commonly in chronic pain syndromes may in fact be limited to males with chronic pain disorders. Future studies are necessary in order to validate and identify possible mechanisms for these observations.

### Microstructural Changes Associated with Pain Severity Changes at 36 Month Follow-Up

Results from the current study appear to demonstrate that long-term (36 month) DTI changes within “disease-associated” areas are associated with pain symptom improvement. Besides decreased MD values observed along certain white matter pathways such as the external capsule, the superior corona radiata, the uncinate and superior longitudinal fasciculi, which were found to be common “disease-associated” areas altered in UCPPS, multiple additional regions were identified as having changed with UCPPS symptom severity over 36 months of observation, consistent with previous studies.^36^

It is worth nothing that the thalamus showed both increasing MD and slightly increasing FA associated with reduction in long-term (36 month) UCPPS pain severity. As the relay center of motor and sensory signals, structural and functional alterations have been consistently reported and discussed within thalamus. Studies have found that changes in fronto-striatal connections may be associated with aversive learning in chronic pain^4^ and dynamic changes between the frontal lobe and striatum might suggest how the possible functional connectivity pattern in fully chronic phase supports pain control and modulation.^26,27^ Taken together, results from the current study may reflect such ongoing structural reorganization associated with an adaptation to chronic pain. Questions remain, however, as to the clinical implications of this adaptation and whether it is potentially reversible with more targeted therapeutic interventions.

### Limitations

Several limitations merit mentioning. The current study assumed that short-term, 6-month interval changes in DTI are not likely to occur despite slight improvement in symptom severity (Fig 5), allowing us to construct “disease-associated” DTI signatures in UCPPS. While it is conceivable that we may be underestimating this effect due to potential *regression of the mean* effects as described by Whitney and Von Korff,^34^ or effects that show symptom improvement subsequent to entering treatment or a clinical trial at least partially due to the patients having high symptom severity when they initially seek treatment, we contest this did not influence our results. First, the MAPP-II trial explicitly addressed potential effects of initial improvement in UCPPS patients through an extended run-in period prior to imaging evaluation.^33^ Second, our assumption that DTI measurements are likely stable over a 6-month period is also supported by the fact that microstructural adaptation occurs over longer periods of time compared with functional alterations, and resting-state fMRI has been used to accurately predict changes in pain symptom severity in the short term, after 3 months, but was not able to predict 6- or 12-month symptom trends.^27^ In line with those findings, associations observed in the current study appeared to suggest that distinct time scales for functional and structural neuroplasticity may occur in UCPPS, with changes in function occurring relatively quickly and changes in structure occurring over longer periods of time. Importantly, the current study defined “disease-associated” DTI signatures based on common differences between UCPPS and healthy controls at the 2 time points and not merely based on symptom severity, so differences in brain microstructure identified in the current study are those that occur even in the presence of slight changes in pain severity. Lastly, Fig 7 illustrating the sensitivity of changes in DTI to changes in pain severity clearly shows a wide variety in pain severity changes for individual patients and clearly indicates the sensitivity of DTI in the regions of interest are not merely based on the subtle *regression of the mean* effect.

Additionally, the number of UCPPS patient was greater than the number of participants in the HC cohort. A larger sample size of HCs would not only improve the statistical power to detect reproducible microstructural differences between the UCPPS and HCs but also help validate that the observed microstructural differences in sex were specific to the UCPPS. Ultimately, the unbalanced nature of MAPP-II consortia study was by design,^7^ as the purpose of MAPP-II was to perform a long-term (36 month) highly integrated and comprehensive cross-modality assessment of clinical, biological, brain imaging, and pain sensitivity measures within a large cohort of UCPPS patients. Furthermore, while the DTI acquisition parameters and sampling scheme were highly standardized and we didn’t identify significant site differences within HCs, it is possible a more robust method for site correction could have been performed like those proposed by Fortin et al.^16^ Moreover, the influence of comorbidities and potential medication on brain plasticity or microstructural alterations in patients with UCPPS were not explicitly considered; however, the influence of medications are likely to be minimal based on lack of brain functional differences between treated and untreated patients.^27^ Also, the HC cohort excluded potential participants if they reported any pain in the pelvic or bladder region or chronic pain in more than one non-urologic body region. Future studies with a better eligibility criterion of healthy controls, analyses accounting for/comparing with other major comorbid conditions (such as fibromyalgia and irritable bowel syndrome, etc) are necessary in determining the imaging biomarker are representative and unique to UCPPS. Lastly, it is possible we could have introduced some error in registration between subjects because we aligned all data to the Johns Hopkins University DTI and Desikan-Killiany cortical atlases and did not use a study population-based template for image registration.

## Conclusions

To summarize, the current longitudinal study identified distinctive FA differences between UCPPS patients and HCs at baseline and 6-month follow-up visits, but reproducible “disease-associated” patterns in altered MD and abnormal microstructural connectivity in UCPPS patients when compared to HCs. These differences were found in regions associated with sensory perception, integration of sensory information and pain modulation. Over longer periods of time, improvement in white matter abnormalities within those regions were associated with improvement in pain symptom severity.

## Supplementary Material

Supplemental Tables

## Figures and Tables

**Table 1.** Cohort Demographics for Diffusion Tensor Imaging and Tractography Analyses

	Group	Race (Ethnicity)	Age & Sex	Med Use	Symptom Duration (years)		Pain Severity	Urinary Severity
UCPPS	N = 364	323 W/41 NW 339 NHisp/25 Hisp	44.1 ± 15.5 [18.4, 78.8] 132M/232F	0: 23 1: 92 2: 162 3: 87	12.0 ± 12.0 [0, 59]	Baseline 6-mo	13.8 ± 5.5 [0, 26] 12.7 ± 5.8 [0, 27]	11.1 ± 5.9 [0, 25] 10.7 ± 5.8 [0, 24]
	N = 166	147 W/19 NW 151 NHisp/15 Hisp	47.7 ± 15.8 [19.3, 78.8] 55M/111F	N/A	13.1 ± 11.8 [0, 52]	36-mo	11.6 ± 6.2 [0, 25]	10.1 ± 5.8 [0, 25]
HCs	N = 61	40 W/21 NW 53 NHisp/8 Hisp	41.8 ± 14.7 [20.8, 73.4] 28M/33F		N/A	Baseline 6-mo	0.2 ± 0.9 [0, 5] 0.5 ± 1.2 [0, 6]	2.7 ± 2.3 [0, 10] 2.5 ± 2.6 [0, 9]

Abbreviations: N, Number; M, Male; F, Female; N/A, Not Applicable; W, White; NW, Non-White; Hisp, Hispanic; NHisp, Non-Hispanic; UCPPS, Urological chronic pelvic pain syndrome; HCs, Healthy controls; Med Use, Medication Use Category; 0, None; 1, Peripheral; 2, Central; 3, Opioid.

**Table 2.** Diffusion Tensor Imaging (DTI) and T1-Weighted Imaging Acquisition Protocols

		Acquisition Protocol #1	Acquisition Protocol #2	Acquisition Protocol #3	Acquisition Protocol #4	Acquisition Protocol #5
Scanning Site		UCLA & UI	UM	NWU	UW	WUSTL
Field Strength & Scanner		3T Siemens Prisma	3T GE Discovery	3T Philips Achieva	3T Siemens Trio	3T Siemens Trio
DTI	Echo Time	88ms	85ms	72ms	88ms	88ms
	Repetition Time	9500ms	9200ms	8400ms	10700ms	9500ms
	Flip Angle	90°	90°	90°	90°	90°
	Matrix Size	128 ± 128	128 ± 128	128 ± 128	128 ± 128	128 ± 128
	Field-of-View	256 ± 256	256 ± 256	256 ± 256	256 ± 256	256 ± 256
	Slice Thickness	2mm	2mm	2mm	2mm	2mm
	Sensitizing Directions	64 Directions	64 Directions	64 Directions	64 Directions	64 Directions
	Diffusion b-value	1,000 s/mm^2^	1,000 s/mm^2^	1,000 s/mm^2^	1,000 s/mm^2^	1,000 s/mm^2^
	Reference b-value	0 s/mm^2^	0 s/mm^2^	0 s/mm^2^	0 s/mm^2^	0 s/mm^2^
T1	Echo Time	2.98ms	3.18ms	3.01ms	2.98ms	2.98ms
	Repetition Time	2300ms	8.15ms	6.64ms	2300ms	2300ms
	Inversion Time	900ms	400ms	0ms	900ms	900ms
	Flip Angle	9°	11°	9°	9°	90
	Matrix Size	240 ± 256	256 ± 256	256 ± 256	240 ± 256	240 ± 256
	Field-of-View	240 ± 256	256 ± 256	256 ± 256	240 ± 256	240 ± 256
	Slice Thickness	1mm	1mm	1mm	1mm	1mm

Abbreviations: UCLA, University of California, Los Angeles; UI, University of Iowa; UM, University of Michigan; NWU, Northwestern University; UW, University of Washington; WUSTL, Washington University in St. Louis (WUSTL).

**Table 3.** White Matter Tracts Showing Significant Differences in Fractional Anisotropy (FA) between UCPPS Patients and HCs at Both Baseline and 6-Month Follow-up.

White Matter Pathways	Baseline		6-mo	
	Overlapping Cluster Size (μL)	UCPPS vs HC	Overlapping Cluster Size (μL)	UCPPS vs HC
Sup Longitudinal Fasciculus l	105	Higher in UCPPS	-	-
Ant Corona Radiata l	80	Higher in UCPPS	-	-
Sup Corona Radiata l	11	Higher in UCPPS	-	-
Ant Corona Radiata r	-	-	244	Higher in HC
External Capsule r	-	-	45	Higher in HC

Abbreviations: Ant, Anterior; Sup, Superior; r, Right; l, Left.

**Table 4.** White Matter Tracts Showing Significant Differences in Mean Diffusivity (MD) between UCPPS Patients and HCs at Both Baseline and 6-Month Follow-up

White Matter Pathways	Baseline		6-Month		Common Cluster Size (μL)
	Overlapping Cluster Size (μL)	UCPPS vs HC	Overlapping Cluster Size (μL)	UCPPS vs HC	
External Capsule l	729	Higher in UCPPS	1121	Higher in UCPPS	379
Thalamus	822	Higher in UCPPS	954	Higher in UCPPS	351
Pos Internal Capsule l	626	Higher in UCPPS	572	Higher in UCPPS	198
Ret Internal Capsule l	369	Higher in UCPPS	312	Higher in UCPPS	184
Sup Corona Radiata l	405	Higher in UCPPS	770	Higher in UCPPS	152
Body Corpus Callosum	201	Higher in UCPPS	731	Higher in UCPPS	123
Pos Corona Radiata l	102	Higher in UCPPS	194	Higher in UCPPS	37
Uncinate Fasciculus l	20	Higher in UCPPS	34	Higher in UCPPS	12
Sup Longitudinal Fasciculus l	44	Higher in UCPPS	238	Higher in UCPPS	9
Pos Thalamic Radiation l	64	Higher in UCPPS	-	-	-
Sup Corona Radiata r	-	-	843	Higher in UCPPS	-
Ant Corona Radiata l	-	-	388	Higher in UCPPS	-
Cingulum Cingulate Gyrus r	-	-	295	Higher in UCPPS	-
Cingulum Cingulate Gyrus l	-	-	218	Higher in UCPPS	-
Ant Internal Capsule l	-	-	117	Higher in UCPPS	-
Fornix Stria Terminalis l	-	-	55	Higher in UCPPS	-
Sup Fronto-occipital Fasciculus l	-	-	49	Higher in UCPPS	-
Pos Corona Radiata r	-	-	29	Higher in UCPPS	-
Sup Longitudinal Fasciculus r	-	-	14	Higher in UCPPS	-

Abbreviations: Ant, Anterior; Sup, Superior; Pos, Posterior; Ret, Retrolenticular Part; r, Right; l, Left.

**Table 5.** White Matter Tracts Showing Significant Correlation with Longitudinal Change of Pain Severity in Fractional Anisotropy (FA) and Mean Diffusivity (MD) Measurements over 36-Month Observation

White Matter Pathways	Fractional Anisotropy		Mean Diffusivity	
	Overlapping Cluster Size (μL)	T-Score	Overlapping Cluster Size (μL)	T-Score
Sup Corona Radiata r	647	2.5612	1316	−2.5925
Thalamus	573	−0.3230	920	−2.5690
Body Corpus Callosum	532	2.5022	1381	−2.5369
Sup Longitudinal Fasciculus r	449	2.5462	610	−2.6333
External Capsule l	186	−2.5315	1072	2.6582
Ant Internal Capsule r	137	2.5972	403	−2.4582
Cingulum Cingulate Gyrus r	136	2.6035	326	−2.4737
Ant Internal Capsule l	89	−2.5164	363	2.5497
Sup Fronto-occipital Fasciculus r	52	3.0180	167	−2.7564
Ant Corona Radiata r	48	−2.3092	67	−2.3300
Sup Corona Radiata l	15	−2.3113	12	2.3291
Splenium Corpus Callosum	359	2.4018	-	-
Pos Internal Capsule l	266	−2.6684	-	-
Cerebral Peduncle l	128	−2.6735	-	-
Ret Internal Capsule l	69	−2.5963	-	-
Fornix	30	2.4839	-	-
Pos Corona Radiata r	23	2.4299	-	-
Sup Fronto-occipital Fasciculus l	16	−2.9352	-	-
Pos Internal Capsule r	-	-	695	−2.6534
External Capsule r	-	-	622	−2.6802
Ant Corona Radiata l	-	-	225	2.4692
Sup Longitudinal Fasciculus l	-	-	161	2.5141
Uncinate Fasciculus l	-	-	122	2.5092
Ret Internal Capsule r	-	-	86	−2.7123
Cingulum Cingulate Gyrus l	-	-	72	−1.0657
Genu Corpus Callosum	-	-	43	2.5611
Sagittal Stratum l	-	-	42	2.6794
Fornix Stria Terminalis l	-	-	15	2.3947

Abbreviations: Ant, Anterior; Sup, Superior; Pos, Posterior; Ret, Retrolenticular Part; r, Right; l, Left.

## Perspective:

This longitudinal study identified reproducible, “disease-associated” patterns in altered mean diffusivity and abnormal microstructural connectivity in UCPPS comparing to HCs over 6 months. These differences were found in regions involved in sensory processing and integration and pain modulation, making it potentially amenable for clinical interventions that target synaptic and/or neuronal reorganization.
