# Supplementary material for: Reproducible Microstructural Changes in the Brain Associated With the Presence and Severity of Urologic Chronic Pelvic Pain Syndrome (UCPPS): A 3-Year Longitudinal Diffusion Tensor Imaging Study From the MAPP Network
Source: J Pain. Author manuscript; Available in PMC 2023 Nov 26. (PMC10676766; doi:10.1016/j.jpain.2022.11.008)
Supplement: Supplemental Tables [file NIHMS1938961-supplement-Supplemental_Tables.docx]

**Reproducible Microstructural Changes in the Brain Associated with the Presence and Severity of Urologic Chronic Pelvic Pain Syndrome (UCPPS): A 3-year Longitudinal Diffusion Tensor Imaging Study from the MAPP Network**

Chencai Wang^1^, Jason J. Kutch^2^, Jennifer S. Labus^3,4,5^, Claire C Yang^6^,

Richard E Harris^7^, Emeran A. Mayer^3,4,5^, Benjamin M. Ellingson^1,5*^

^1^Department of Radiological Science, David Geffen School of Medicine, University of California Los Angeles, Los Angeles, CA

^2^Division of Biokinesiology and Physical Therapy, University of Southern California, Los Angeles, CA

^3^Oppenheimer Center for the Neurobiology of Stress, and PAIN, David Geffen School of Medicine, University of California Los Angeles, Los Angeles, CA

^4^Department of Digestive Diseases and Gastroenterology, David Geffen School of Medicine, University of California Los Angeles, Los Angeles, CA

^5^Department of Psychiatry and Biobehavioral Sciences, David Geffen School of Medicine, University of California Los Angeles, Los Angeles, CA

^6^Department of Urology, University of Washington, Seattle, Washington USA

^7^Chronic Pain and Fatigue Research Center, Department of Anesthesiology, University of Michigan, Ann Arbor, MI, USA

**Table S1.** Cohort Demographics for Diffusion Tensor Imaging and Tractography Analyses of Each Site.

|  | **Group** | **Race (Ethnicity)** | **Age & Sex** | **Med Use** | **Symptom Duration (years)** |  | **Pain**  **Severity** | **Urinary**  **Severity** |
| --- | --- | --- | --- | --- | --- | --- | --- | --- |
| **UCPPS** | Site #1  N=45 | 31 W/14 NW  40 NHisp/5 Hisp | 44.8 $\pm$ 14.4  [19.5, 78.4]  18M/27F | 0: 10  1: 12  2: 18  3: 5 | 11.4 $\pm$ 12.1  [0, 43] | **Baseline** | 12.3 $\pm$ 5.5  [2, 24] | 10.6 $\pm$ 6.3  [1, 24] |
|  |  |  |  |  |  | **6-Month** | 11.6 $\pm$ 5.3  [0, 25] | 10.3 $\pm$ 5.9  [1, 22] |
|  | Site #2  N=59 | 50 W/9 NW  54 NHisp/5 Hisp | 39.8 $\pm$ 15.5  [19.3, 78.0]  16M/43F | 0: 4  1: 16  2: 21  3: 18 | 12.1 $\pm$ 12.0  [0, 47] | **Baseline** | 15.3 $\pm$ 4.9  [3, 26] | 11.8 $\pm$ 6.4  [0, 25] |
|  |  |  |  |  |  | **6-Month** | 13.8 $\pm$ 5.3  [2, 26] | 11.1 $\pm$ 6.3  [0, 24] |
|  | Site #3  N=76 | 75 W/1 NW  75 NHisp/1 Hisp | 49.8 $\pm$ 16.5  [19.7, 78.8]  20M/56F | 0: 0  1: 13  2: 43  3: 20 | 12.7 $\pm$ 11.5  [1, 57] | **Baseline** | 13.2 $\pm$ 5.2  [2, 26] | 11.5 $\pm$ 5.7  [1, 24] |
|  |  |  |  |  |  | **6-Month** | 11.9 $\pm$ 5.9  [0, 27] | 11.2 $\pm$ 5.4  [2, 24] |
|  | Site #4  N=58 | 51 W/7 NW  53 NHisp/5 Hisp | 40.8 $\pm$ 14.6  [19.4, 65.6]  16M/42F | 0: 2  1: 23  2: 23  3: 10 | 12.9 $\pm$ 13.6  [1, 59] | **Baseline** | 15.0 $\pm$ 5.3  [0, 24] | 12.1 $\pm$ 5.8  [1, 22] |
|  |  |  |  |  |  | **6-Month** | 14.1 $\pm$ 5.8  [0, 23] | 11.6 $\pm$ 5.4  [2, 22] |
|  | Site #5  N=57 | 52 W/5 NW  51 NHisp/6 Hisp | 46.2 $\pm$ 15.7  [23.5, 78.1]  38M/19F | 0: 4  1: 18  2: 18  3: 17 | 11.5 $\pm$ 13.6  [0, 54] | **Baseline** | 12.2 $\pm$ 5.5  [0, 23] | 9.4 $\pm$ 6.3  [0, 23] |
|  |  |  |  |  |  | **6-Month** | 12.4 $\pm$ 5.5  [0, 26] | 9.5 $\pm$ 6.2  [0, 23] |
|  | Site #6  N=69 | 64 W/5 NW  66 NHisp/3 Hisp | 43.0 $\pm$ 14.2  [18.4, 71.4]  24M/45F | 0: 3  1: 10  2: 39  3: 17 | 11.5 $\pm$ 9.6  [1, 45] | **Baseline** | 14.2 $\pm$ 6.0  [0, 25] | 11.1 $\pm$ 5.6  [1, 24] |
|  |  |  |  |  |  | **6-Month** | 12.3 $\pm$ 6.0  [0, 24] | 10.5 $\pm$ 5.7  [2, 23] |
|  | N=364 | 323 W/41 NW  339 NHisp/25 Hisp | 44.1 $\pm$ 15.5  [18.4, 78.8]  132M/232F | 0: 23  1: 92  2: 162  3: 87 | 12.0 $\pm$ 12.0  [0, 59] | **Baseline** | 13.8 $\pm$ 5.5  [0, 26] | 11.1 $\pm$ 5.9  [0, 25] |
|  |  |  |  |  |  | **6-Month** | 12.7 $\pm$ 5.8  [0, 27] | 10.7 $\pm$ 5.8  [0, 24] |
| **HCs** | Site #1  N=10 | 5 W/5 NW  8 NHisp/2 Hisp | 42.7 $\pm$ 16.0  [24.8, 63.9]  5M/5F | N/A | N/A | **Baseline** | 0.4 $\pm$ 1.3  [0, 4] | 2.3 $\pm$ 2.0  [0, 6] |
|  |  |  |  |  |  | **6-Month** | 0 | 1.4 $\pm$ 1.8  [0, 6] |
|  | Site #2  N=11 | 6 W/5 NW  9 NHisp/2 Hisp | 41.6 $\pm$ 12.1  [23.4, 56.8]  6M/5F | N/A | N/A | **Baseline** | 0 | 2.9 $\pm$ 1.8  [0, 6] |
|  |  |  |  |  |  | **6-Month** | 0 | 1.7 $\pm$ 1.7  [0, 5] |
|  | Site #3  N=10 | 10 W/0 NW  10 NHisp/0 Hisp | 47.5 $\pm$ 16.1  [22.7, 73.4]  4M/6F | N/A | N/A | **Baseline** | 0.5 $\pm$ 1.6  [0, 5] | 3.0 $\pm$ 2.2  [0, 7] |
|  |  |  |  |  |  | **6-Month** | 1.1 $\pm$ 2.3  [0, 6] | 2.8 $\pm$ 2.5  [0, 9] |
|  | Site #4  N=10 | 7 W/3 NW  9 NHisp/1 Hisp | 36.3 $\pm$ 13.1  [21.9, 59.1]  5M/5F | N/A | N/A | **Baseline** | 0 | 3.2 $\pm$ 2.9  [0, 10] |
|  |  |  |  |  |  | **6-Month** | 0.1 $\pm$ 0.3  [0, 1] | 4.1 $\pm$ 3.5  [0, 9] |
|  | Site #5  N=8 | 4 W/4 NW  5 NHisp/3 Hisp | 37.8 $\pm$ 13.9  [21.7, 61.3]  4M/4F | N/A | N/A | **Baseline** | 0 | 3.0 $\pm$ 3.5  [0, 9] |
|  |  |  |  |  |  | **6-Month** | 1.0 $\pm$ 1.5  [0, 4] | 3.8 $\pm$ 2.9  [0, 8] |
|  | Site #6  N=12 | 8 W/4 NW  12 NHisp/0 Hisp | 43.6 $\pm$ 17.3  [20.8, 67.3]  4M/8F | N/A | N/A | **Baseline** | 0.3 $\pm$ 0.7  [0, 2] | 2.1 $\pm$ 2.3  [0, 7] |
|  |  |  |  |  |  | **6-Month** | 0.6 $\pm$ 1.2  [0, 3] | 2.1 $\pm$ 2.2  [0, 8] |
|  | N=61 | 40 W/21 NW  53 NHisp/8 Hisp | 41.8 $\pm$ 14.7  [20.8, 73.4]  28M/33F | N/A | N/A | **Baseline** | 0.2 $\pm$ 0.9  [0, 5] | 2.7 $\pm$ 2.3  [0, 10] |
|  |  |  |  |  |  | **6-Month** | 0.5 $\pm$ 1.2  [0, 6] | 2.5 $\pm$ 2.6  [0, 9] |
| **36-Month**  **UCPPS** | Site #1  N=22 | 15 W/7 NW  19 NHisp/3 Hisp | 47.3 $\pm$ 15.4  [19.5, 78.4]  7M/15F | 0: 6  1: 5  2: 6  3: 5 | 10.5 $\pm$ 10.6  [1, 43] | **36-Month** | 10.9 $\pm$ 5.3  [2, 20] | 10.4 $\pm$ 5.3  [4, 20] |
|  | Site #2  N=19 | 13 W/6 NW  15 NHisp/4 Hisp | 37.9 $\pm$ 15.7  [19.3, 78.0]  5M/14F | 0: 2  1: 3  2: 6  3: 8 | 12.2 $\pm$ 11.3  [1, 37] | **36-Month** | 13.4 $\pm$ 4.9  [6, 24] | 11.4 $\pm$ 5.9  [0, 21] |
|  | Site #3  N=42 | 42 W/0 NW  41 NHisp/1 Hisp | 54.0 $\pm$ 16.0  [25.3, 78.8]  13M/29F | 0: 0  1:8  2: 25  3: 9 | 13.8 $\pm$ 10.6  [1, 49] | **36-Month** | 10.0 $\pm$ 6.9  [0, 25] | 9.6 $\pm$ 6.0  [0, 22] |
|  | Site #4  N=21 | 17 W/4 NW  17 NHisp/4 Hisp | 44.1 $\pm$ 15.8  [19.8, 65.2]  5M/16F | 0: 0  1: 9  2: 8  3: 4 | 13.8 $\pm$ 13.4  [3, 50] | **36-Month** | 13.9 $\pm$ 5.9  [0, 23] | 12.1 $\pm$ 5.7  [2, 21] |
|  | Site #5  N=29 | 28 W/1 NW  28 NHisp/1 Hisp | 47.6 $\pm$ 17.5  [25.4, 78.1]  11M/18F | 0: 0  1: 12  2: 11  3: 6 | 12.3 $\pm$ 13.1  [0, 52] | **36-Month** | 11.7 $\pm$ 5.8  [0, 23] | 10.6 $\pm$ 6.4  [0, 25] |
|  | Site #6  N=33 | 32 W/1 NW  31 NHisp/2 Hisp | 47.0 $\pm$ 12.8  [23.2, 69.1]  14M/19F | 0: 3  1: 6  2: 16  3: 8 | 12.9 $\pm$ 10.9  [2, 45] | **36-Month** | 10.9 $\pm$ 6.5  [0, 25] | 9.0 $\pm$ 5.6  [1, 22] |
|  | N=166 | 147 W/19 NW  151 NHisp/15 Hisp | 47.7 $\pm$ 15.8  [19.3, 78.8]  55M/111F | 0: 11  1: 43  2: 72  3: 40 | 13.1 $\pm$ 11.8  [0, 52] | **36-Month** | 11.6 $\pm$ 6.2  [0, 25] | 10.1 $\pm$ 5.8  [0, 25] |

N=Number, M=Male, F=Female, N/A=Not Applicable

W=White, NW=Non-White, Hisp=Hispanic, NHisp=Non-Hispanic

UCPPS= Urological chronic pelvic pain syndrome, HCs=Healthy controls

Med Use=Medication Use Category, 0=None, 1=Peripheral, 2=Central, 3=Opioid

**Table S2.** Abbreviations for the Cortical and Subcortical Regions of Interest (ROIs).

| **Lobes of the Brain** | **Regions of Interest (ROIs)** | **Abbreviations** |
| --- | --- | --- |
| **Prefrontal & Frontal**  **Lobe** | **Frontal Pole** | FP |
|  | **Superior Frontal Gyrus** | SFG |
|  | **Rostral Middle Frontal Gyrus** | RostMFG |
|  | **Caudal Middle Frontal Gyrus** | CaudMFG |
|  | **Inferior Frontal Gyrus, Pars Triangularis** | IFG tri |
|  | **Inferior Frontal Gyrus, Pars Opercularis** | IFG oper |
|  | **Inferior Frontal Gyrus, Pars Orbitalis** | IFG orb |
|  | **Medial Orbitofrontal Gyrus** | MedOFC |
|  | **Rostral Anterior Cingulate Cortex** | RostAC |
|  | **Caudal Anterior Cingulate Cortex** | CaudAC |
|  | **Lateral Orbitofrontal Gyrus** | LOFG |
| **Primary Sensorimotor Lobe** | **Precentral Gyrus** | PreCG |
|  | **Postcentral Gyrus** | PostCG |
|  | **Paracentral Gyrus** | ParaCG |
| **Temporal**  **Lobe** | **Temporal Pole** | TP |
|  | **Superior Temporal Gyrus** | STG |
|  | **Middle Temporal Gyrus** | MTG |
|  | **Inferior Temporal Gyrus** | ITG |
|  | **Fusiform Gyrus** | FusG |
|  | **Heschl’s Gyrus** | HG |
|  | **Entorhinal Cortex** | EC |
| **Parietal**  **Lobe** | **Superior Parietal Lobule** | SPL |
|  | **Supramarginal Gyrus** | SMG |
|  | **Inferior Parietal Lobule** | IPL |
|  | **Precuneus** | Precuneus |
| **Occipital**  **Lobe** | **Lateral Occipital Cortex** | LOC |
|  | **Pericalcarine Cortex** | PerCC |
|  | **Cuneus** | Cuneus |
|  | **Lingual Gyrus** | LG |
| **Limbic**  **System** | **Posterior Cingulate Cortex** | PC |
|  | **Isthmus of Cingulate Cortex** | IsthC |
|  | **Parahippocampus** | ParaHCP |
|  | **Hippocampus** | HCP |
|  | **Insular Cortex** | Insula |
| **Thalamus &**  **Basal Ganglia** | **Thalamus** | Thalamus |
|  | **Caudate** | Caudate |
|  | **Putamen** | Putamen |
|  | **Pallidum** | Pallidum |
| **Subcortex** | **Cerebellum** | Cerebellum |
|  | **Amygdala** | Amygdala |
|  | **Accumbens** | Accumbens |
|  | **Brainstem** | Brainstem |

**Table S3. White Matter Tracts Showing Significant Differences in Fractional Anisotropy (FA) between Male and Female UCPPS Patients at Both Baseline and 6-Month Follow-up.**

| **White Matter Pathways** | **Baseline** | | **6-Month** | | **Common Cluster**  **Size (**$\boldsymbol{\mu L}$**)** |
| --- | --- | --- | --- | --- | --- |
|  | **Overlapping**  **Cluster**  **Size (**$\boldsymbol{\mu L}$**)** | **Male**  **vs**  **Female** | **Overlapping**  **Cluster**  **Size (**$\boldsymbol{\mu L}$**)** | **Male**  **vs**  **Female** |  |
| **Ant Internal Capsule l** | 353 | Female | 330 | Male | 178 |
| **Ant Internal Capsule r** | 473 | Male | 321 | Male | 144 |
| **Ant Corona Radiata l** | 3205 | Female | 3295 | Female | 2816 |
| **Ant Corona Radiata r** | 1823 | Female | 1593 | Female | 1230 |
| **Body Corpus Callosum** | 3337 | Female | 2763 | Female | 2056 |
| **Cerebral Peduncle l** | 622 | Female | 331 | Male | 226 |
| **Cerebral Peduncle r** | 376 | Male | 520 | Male | 227 |
| **Cingulum Cingulate Gyrus l** | 788 | Male | 874 | Male | 681 |
| **Cingulum Cingulate Gyrus r** | 608 | Male | 558 | Male | 390 |
| **Corticospinal Tract l** | 384 | Male | 408 | Male | 317 |
| **Corticospinal Tract r** | 411 | Male | 443 | Male | 333 |
| **External Capsule l** | 1338 | Male | 1134 | Male | 831 |
| **External Capsule r** | 1193 | Male | 879 | Male | 639 |
| **Fornix** | 285 | Female | 278 | Female | 217 |
| **Fornix Stria Terminalis l** | 311 | Female | 283 | Female | 225 |
| **Fornix Stria Terminalis r** | 237 | Female | 254 | Female | 182 |
| **Genu Corpus Callosum** | 1408 | Female | 1412 | Female | 975 |
| **Inf Cerebellar Peduncle l** | 266 | Male | 217 | Male | 170 |
| **Inf Cerebellar Peduncle r** | 352 | Male | 254 | Male | 209 |
| **Medial Lemniscus l** | 284 | Male | 231 | Male | 205 |
| **Medial Lemniscus r** | 319 | Male | 222 | Male | 207 |
| **Mid Cerebellar Peduncle** | 3138 | Male | 3656 | Female | 2078 |
| **Pontine Crossing Tract** | 74 | Male | 140 | Male | 54 |
| **Pos Internal Capsule l** | 778 | Male | 834 | Male | 509 |
| **Pos Internal Capsule r** | 841 | Male | 929 | Female | 538 |
| **Pos Corona Radiata l** | 1050 | Female | 910 | Female | 639 |
| **Pos Corona Radiata r** | 574 | Female | 493 | Female | 362 |
| **Pos Thalamic Radiation l** | 1587 | Female | 1625 | Female | 1313 |
| **Pos Thalamic Radiation r** | 1169 | Female | 1000 | Female | 784 |
| **Ret Internal Capsule l** | 550 | Female | 654 | Female | 415 |
| **Ret Internal Capsule r** | 122 | Female | 166 | Female | 59 |
| **Sagittal Stratum l** | 650 | Female | 652 | Female | 507 |
| **Sagittal Stratum r** | 486 | Female | 360 | Female | 259 |
| **Splenium Corpus Callosum** | 2238 | Female | 1852 | Female | 1049 |
| **Sup Cerebellar Peduncle l** | 415 | Male | 312 | Male | 234 |
| **Sup Cerebellar Peduncle r** | 414 | Male | 433 | Male | 326 |
| **Sup Corona Radiata l** | 2293 | Female | 2070 | Female | 1702 |
| **Sup Corona Radiata r** | 1814 | Female | 1610 | Female | 1122 |
| **Sup Fronto-occipital Fasciculus l** | 108 | Female | 147 | Female | 87 |
| **Sup Fronto-occipital Fasciculus r** | 97 | Male | 112 | Female | 62 |
| **Sup Longitudinal Fasciculus l** | 1037 | Female | 1248 | Male | 652 |
| **Sup Longitudinal Fasciculus r** | 748 | Male | 869 | Male | 545 |
| **Tapetum l** | 129 | Female | 136 | Female | 109 |
| **Tapetum r** | 241 | Female | 238 | Female | 208 |
| **Uncinate Fasciculus r** | 193 | Male | 192 | Male | 171 |
| **Thalamus** | 7639 | Female | 7125 | Male | 4863 |

Ant=Anterior, Pos=Posterior, Sup=Superior, Pos=Posterior

Ret=Retrolenticular Part, r=Right, l=Left.

**Table S4. White Matter Tracts Showing Significant Differences in Mean Diffusivity (MD) between Male and Female UCPPS Patients at Both Baseline and 6-Month Follow-up.**

| **White Matter Pathways** | **Baseline** | | **6-Month** | | **Common Cluster**  **Size (**$\boldsymbol{\mu L}$**)** |
| --- | --- | --- | --- | --- | --- |
|  | **Overlapping**  **Cluster**  **Size (**$\boldsymbol{\mu L}$**)** | **Male**  **vs**  **Female** | **Overlapping**  **Cluster**  **Size (**$\boldsymbol{\mu L}$**)** | **Male**  **vs**  **Female** |  |
| **Ant Internal Capsule l** | 266 | Female | 173 | Female | 106 |
| **Ant Internal Capsule r** | 368 | Female | 239 | Female | 160 |
| **Ant Corona Radiata l** | 1849 | Male | 3011 | Male | 1650 |
| **Ant Corona Radiata r** | 1681 | Male | 2099 | Male | 1505 |
| **Body Corpus Callosum** | 1361 | Male | 2209 | Male | 889 |
| **Cerebral Peduncle l** | 466 | Female | 415 | Female | 256 |
| **Cerebral Peduncle r** | 1014 | Female | 947 | Female | 755 |
| **Cingulum Cingulate Gyrus l** | 730 | Female | 686 | Female | 400 |
| **Cingulum Cingulate Gyrus r** | 411 | Female | 727 | Female | 262 |
| **Corticospinal Tract l** | 438 | Female | 468 | Female | 365 |
| **Corticospinal Tract r** | 551 | Female | 607 | Female | 466 |
| **External Capsule r** | 384 | Female | 425 | Female | 176 |
| **Fornix** | 477 | Male | 497 | Male | 457 |
| **Fornix Stria Terminalis l** | 217 | Male | 253 | Male | 184 |
| **Fornix Stria Terminalis r** | 433 | Male | 439 | Male | 368 |
| **Genu Corpus Callosum** | 1404 | Male | 2419 | Male | 1260 |
| **Inf Cerebellar Peduncle l** | 202 | Female | 87 | Female | 67 |
| **Inf Cerebellar Peduncle r** | 215 | Female | 104 | Female | 83 |
| **Medial Lemniscus l** | 100 | Female | 117 | Female | 76 |
| **Medial Lemniscus r** | 200 | Female | 80 | Female | 71 |
| **Mid Cerebellar Peduncle** | 5293 | Female | 4910 | Female | 3640 |
| **Pontine Crossing Tract** | 991 | Female | 896 | Female | 771 |
| **Pos Internal Capsule l** | 997 | Female | 423 | Female | 298 |
| **Pos Internal Capsule r** | 1071 | Female | 353 | Female | 284 |
| **Pos Corona Radiata l** | 1941 | Male | 1750 | Male | 1481 |
| **Pos Corona Radiata r** | 1783 | Male | 1987 | Male | 1471 |
| **Pos Thalamic Radiation l** | 1453 | Male | 1320 | Male | 1014 |
| **Pos Thalamic Radiation r** | 1317 | Male | 882 | Male | 740 |
| **Ret Internal Capsule l** | 722 | Male | 455 | Male | 368 |
| **Ret Internal Capsule r** | 358 | Male | 252 | Male | 218 |
| **Sagittal Stratum l** | 680 | Male | 717 | Male | 463 |
| **Sagittal Stratum r** | 583 | Male | 575 | Male | 393 |
| **Splenium Corpus Callosum** | 1544 | Male | 1314 | Male | 649 |
| **Sup Cerebellar Peduncle l** | 259 | Female | 257 | Female | 173 |
| **Sup Cerebellar Peduncle r** | 235 | Female | 172 | Female | 131 |
| **Sup Corona Radiata l** | 2305 | Male | 1911 | Male | 1495 |
| **Sup Corona Radiata r** | 775 | Male | 769 | Male | 397 |
| **Sup Fronto-occipital Fasciculus l** | 175 | Male | 201 | Male | 162 |
| **Sup Fronto-occipital Fasciculus r** | 109 | Male | 142 | Male | 97 |
| **Sup Longitudinal Fasciculus l** | 648 | Female | 582 | Male | 308 |
| **Sup Longitudinal Fasciculus r** | 325 | Male | 212 | Male | 164 |
| **Tapetum l** | 64 | Male | 68 | Male | 52 |
| **Tapetum r** | 159 | Male | 152 | Male | 133 |
| **Uncinate Fasciculus r** | 180 | Female | 196 | Female | 156 |
| **Thalamus** | 9690 | Male | 7937 | Male | 6454 |

Ant=Anterior, Pos=Posterior, Sup=Superior, Pos=Posterior

Ret=Retrolenticular Part, r=Right, l=Left.

**
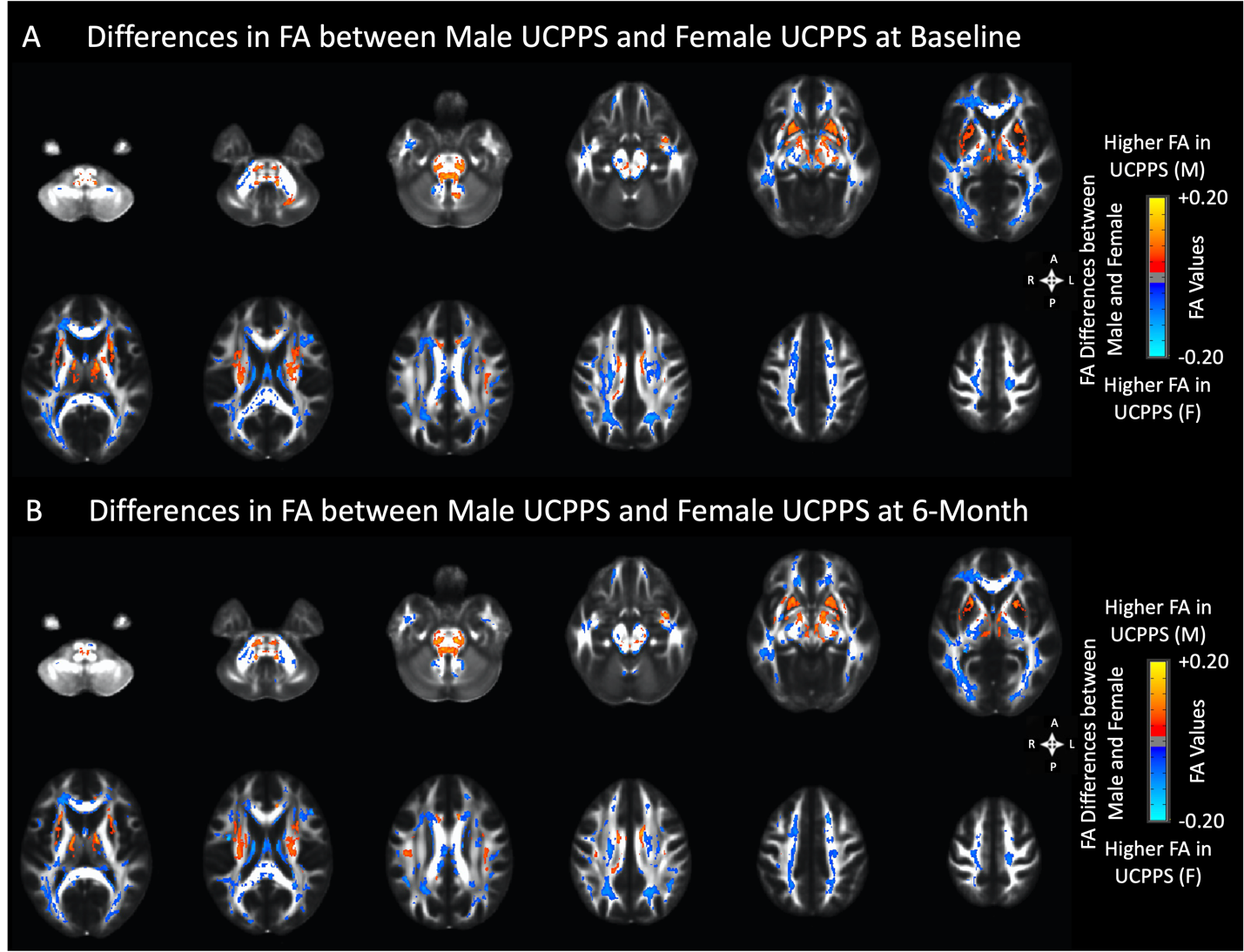
**

**Fig. S1.** Anatomical localization of significant differences in fractional anisotropy (FA) between male UCPPS patients and female UCPPS patients A) at baseline (axial view), and B) at 6-month follow-up (axial view). Significant regions were determined by thresholding based on level of statistical significance, *p* < 0.05, and level of family-wise error (FWE) *α* < 0.05.

**
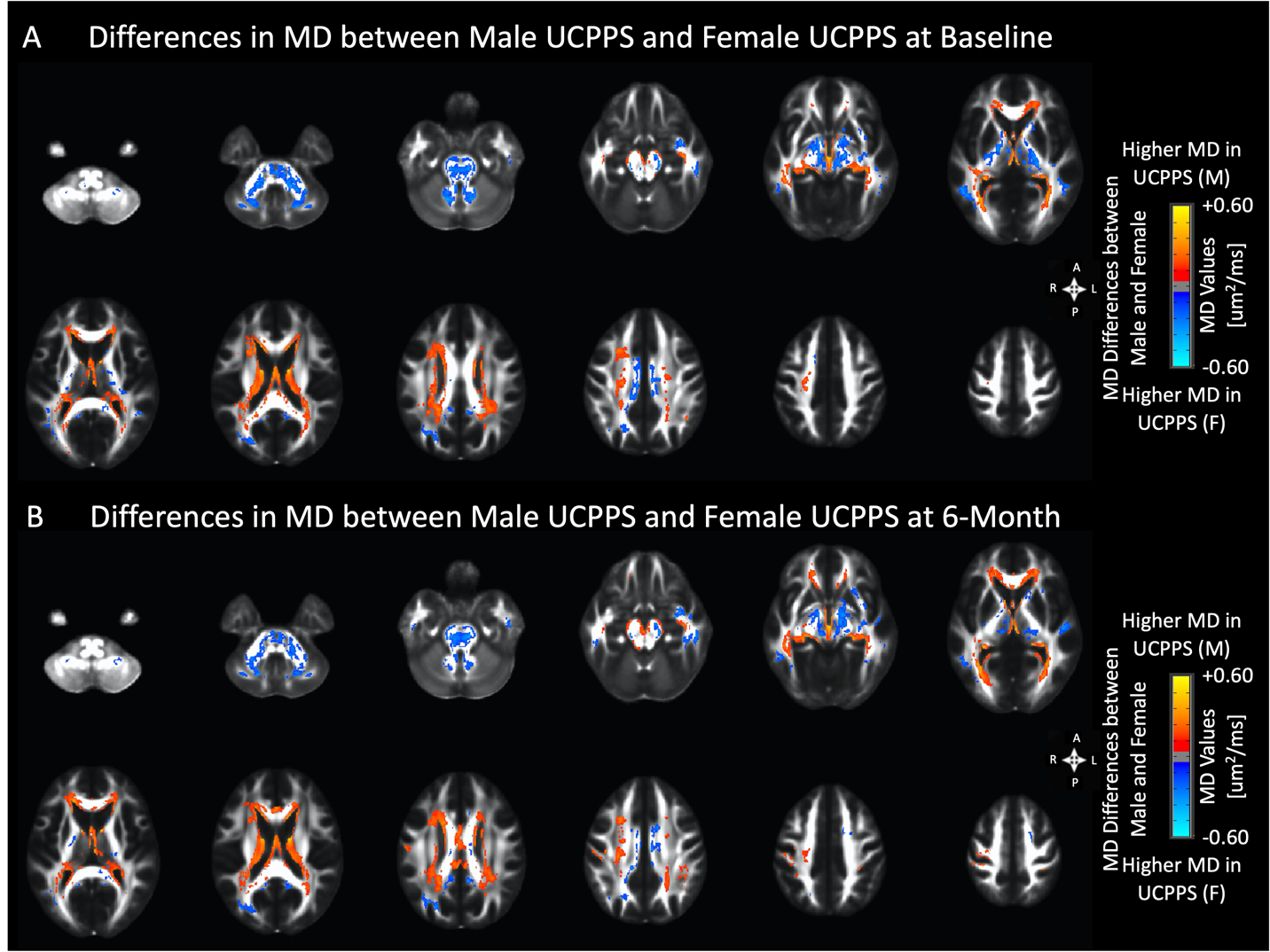
**

**Fig. S2.** Anatomical localization of significant differences in mean diffusivity (MD) between male UCPPS patients and female UCPPS patients A) at baseline (axial view), and B) at 6-month follow-up (axial view). Significant regions were determined by thresholding based on level of statistical significance, *p* < 0.05, and level of family-wise error (FWE) *α* < 0.05.
